# Prediction of BOD, COD, and Total Nitrogen Concentrations in a Typical Urban River Using a Fluorescence Excitation-Emission Matrix with PARAFAC and UV Absorption Indices

**DOI:** 10.3390/s120100972

**Published:** 2012-01-16

**Authors:** Jin Hur, Jinwoo Cho

**Affiliations:** Department of Environment & Energy, Sejong University, 98 Gunja-dong, Gwangjin-ku, Seoul 143-747, Korea; E-Mail: jinwoocho@sejong.ac.kr

**Keywords:** fluorescence spectroscopy, parallel factor analysis, water quality monitoring, urban river, multiple regression analysis

## Abstract

The development of a real-time monitoring tool for the estimation of water quality is essential for efficient management of river pollution in urban areas. The Gap River in Korea is a typical urban river, which is affected by the effluent of a wastewater treatment plant (WWTP) and various anthropogenic activities. In this study, fluorescence excitation-emission matrices (EEM) with parallel factor analysis (PARAFAC) and UV absorption values at 220 nm and 254 nm were applied to evaluate the estimation capabilities for biochemical oxygen demand (BOD), chemical oxygen demand (COD), and total nitrogen (TN) concentrations of the river samples. Three components were successfully identified by the PARAFAC modeling from the fluorescence EEM data, in which each fluorophore group represents microbial humic-like (C1), terrestrial humic-like organic substances (C2), and protein-like organic substances (C3), and UV absorption indices (UV_220_ and UV_254_), and the score values of the three PARAFAC components were selected as the estimation parameters for the nitrogen and the organic pollution of the river samples. Among the selected indices, UV_220_, C3 and C1 exhibited the highest correlation coefficients with BOD, COD, and TN concentrations, respectively. Multiple regression analysis using UV_220_ and C3 demonstrated the enhancement of the prediction capability for TN.

## 1. Introduction

Continuous water quality monitoring is essential for efficient management of urban rivers and for the prompt control of pollution. Due to the rapid responses of urban rivers to intensive land use and/or diverse pollution sources, the deterioration of the water quality may be accelerated, immediately posing a direct or indirect threat to human health and aquatic ecosystems [1–3]. The degree of organic pollution which occurs due to an excessive amount of organic matter, has typically been monitored by measuring BOD and COD values in rivers. A high level of BOD deteriorates river water quality by rapid decomposition of biodegradable organic matter and the subsequent depletion of dissolved oxygen, while COD traditionally represents the total organic matter. However, both concentrations are quantified by the amount of oxygen consumed for a particular chemical oxidation of organic compounds in samples. Enrichment of total nitrogen in urban rivers may result in excessive growth of algae and macrophytes, decreased biodiversity, and odor problems [3,4].

There are several limitations of the traditional water quality parameters for use in continuous monitoring. For example, at least five days are required for the completion of the BOD measurements. The presence of toxic substances may influence the biochemical oxidation, resulting in analytical errors. Potassium dichromate, a typical oxidant for a COD test, cannot completely decompose organic matter in samples, and the degree of the chemical oxidation itself may be affected by organic matter composition and the molecular structures involved [5]. Therefore, it is necessary to develop more rapid and reliable monitoring techniques to replace the traditional water quality parameter measurements. The developed techniques are also expected to serve as a pre-screening tool to select the intensive monitoring sites for extensive urban river systems.

Biosensors based on variations in currents produced from oxygen-consuming microorganisms have been suggested as a continuous monitoring tool for organic pollution. However, their short lifetime and vulnerability to environmental factors limit their applications for continuous monitoring [6]. Recently, fluorescence spectroscopy has emerged as a useful optical sensor-based monitoring technique for organic pollution [1,7,8]. In particular, EEM spectroscopy can capture various fluorescent components contained in water samples over a wide range of excitation and emission wavelengths. In EEM, protein-like fluorescence peaks have been linked to the amount of microbially produced aromatic amino acids such as tryptophan and tyrosine, while humic-like fluorescence peaks have been assigned to the presence of condensed humified organic materials [8,9]. Hudson *et al.* [10] have shown that a tryptophan-like fluorescence peak designated at excitation/emission wavelengths of 275/340 nm could be used as an indicator of the amount of biodegradable organic matters in river water samples. Despite its usefulness as a monitoring tool, however, most of the EEM studies simply relied on “peak picking” to quantify the desired fluorescent components, neglecting the potential bias related to the spectral overlaps from a complex mixture of different fluorophores.

PARAFAC, a three way-decomposition method, has been found to be very useful in identifying the independent spectra of different types of fluorophores [11]. It is advantageous to use it as a monitoring technique beyond fluorescence EEM because it can track even small variations in EEM datasets by separating several independent groups of fluorophores from the overlapped components with a high resolution. In contrast, the weakness of PARAFAC model may include the assumption of the independence among the estimated components in the model, and potential inclusion of one or more poorly estimated components, which may significantly affect the spectra and scores of all other components [12]. Most of the previous studies using PARAFAC for environmental monitoring have focused on tracking organic matter sources and/or characterizing organic matter composition [13–15]. Despite the strong implications of the close association between the fluorescence components extracted by PARAFAC and the degree of water pollution especially expressed by BOD and COD concentrations, only a few related studies have been reported [16]. Furthermore, potential applications of the PARAFAC modeling for estimating water quality parameters in typical urban rivers, which are affected by treated sewage and various anthropogenic activities, have not been fully explored. Therefore, the objectives of this study were: (1) to examine spatial variations in DOM fluorescence characteristics of a typical urban river using fluorescence EEM-PARAFAC; and (2) to estimate the degree of organic pollution and total nitrogen concentrations based on the correlations between BOD, COD, TN concentrations and the PARAFAC components.

## 2. Experimental Section

### 2.1. Study Area and Sample Collection

**Figure 1.**
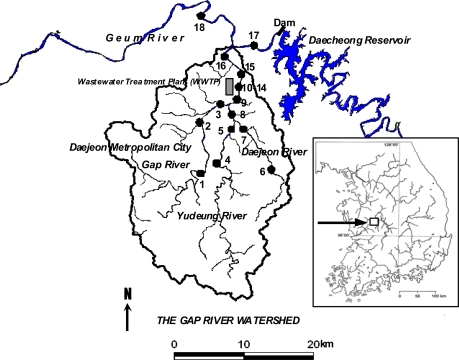
The Gap River watershed. Filled circles indicate sampling locations (adopted from Hur *et al*. [8]).

Water samples were collected in September and October, 2005 (09/25/2005, 10/18/2005) from eighteen locations of the Gap River watershed, a typical urban river flowing through the city of Daejeon, Korea (36°20′N, 127°26′E) with a population of 920,000. The catchment area of the watershed is 662 km^2^ and the land use is 58% forest, 22% agriculture, and 20% urban. A municipal WWTP with a treatment capacity of 900,000 ton/day is located at the middle of the main channel of Gap River (Figure 1).

The water quality downstream of the WWTP is deteriorated by the effluent. There are two tributaries of Gap River called Yudeung River (St. 4 and 5) and Daejeon River (St. 6, 7, 8), which are located upstream of the WWTP but still affected by urban anthropogenic activities (Figure 1). Discharge from a dam reservoir (St. 17) finally joins the main channel of Gap River downstream of the WWTP. A more detailed description of the sampling locations is provided elsewhere [8]. Collected samples were kept refrigerated during transport in field before they were analyzed in a laboratory.

### 2.2. Analytical Methods

Turbidity, temperature, and pH were recorded at the sampling sites. All other analyses were made within one week after the sample collection. The collected samples were first filtered through a 0.1 mm mesh sieve to remove large sized suspended solids. The concentrations of BOD, COD, TN, and total suspended solid (TSS) were determined according to the corresponding standard methods [17]. An aliquot (50 mL) of the samples was passed through a pre-ashed GF/F filter and they were acidified with 1 M HCl to pH 3.0 for the measurements of dissolved organic carbon (DOC) and fluorescence EEM.

DOC concentrations of the samples were determined by a Shimadzu V-CPH analyzer. The relative precision of DOC measurements was <3% based on repeated measurements. Absorption measurements were performed on Varian Cary 300 Conc UV-visible spectrophotometer in a 1 cm quartz cuvette. The aliquots of the samples were diluted prior to the fluorescence measurements until UV absorbance at 254 nm was below 0.05/cm to avoid the inner-filter correction [8,18]. Fluorescence EEM of the diluted samples were generated using a luminescence spectrometer (LS-55, Perkin-Elmer) by scanning emission spectra from 300 to 550 nm at 0.5 nm increments by varying the excitation wavelength from 250 to 400 nm at 5 nm increments. Excitation and emission slits were adjusted to 10 nm and 5 nm, respectively, and the scanning speed was set at 1,200 nm/min. To limit second order Raleigh scattering, a 290 nm cutoff filter was used for all the samples. No shift of fluorescence peaks was observed by comparing the emission spectra with and without inner-filter correction for this study [8]. The fluorescence response to a blank solution (Milli-Q water) was subtracted from the EEM of each sample [19]. The measured fluorescence intensities were then standardized to a Raman peak at 395 nm emission following a suggestion by Baker [7]. Relative precisions of <2% were routinely obtained by three-times repeated fluorescence measurements of randomly chosen field samples.

### 2.3. PARAFAC Modeling

For this study, PARAFAC was applied to fully utilize the fluorescence EEM data of the samples. PARAFAC decomposes the EEM dataset into a set of trilinear terms and a residual array [18] and it estimates the underlying EEM spectra by minimizing the sum of squared residual of the trilinear model:

(1) $$x_{\mathrm{ijk}}=\sum_{f=1}^{F}a_{\mathrm{if}}\;b_{\mathrm{jf}}\;c_{\mathrm{kf}}+\varepsilon_{\mathrm{ijk}}\;\;i=1,...,I;\;\;j=I,...,J;\;\;k=1,...,K$$

where x*_ijk_* is the intensity of fluorescence for the *i*th sample at emission wavelength *j* and excitation wavelength *k*. a*_if_* is directly proportional to the concentration of the *f*th fluorophore in the *i*th sample (defined as scores). b*_jf_* and c*_kf_* are the estimates of the emission and excitation spectra, respectively, for the *f*th fluorophore. F represents the number of components in the model and ε*_ijk_* is the residual element, representing the variability not accounted for by the model [11].

PARAFAC modeling was performed using the MATLAB 7.0 (Mathworks, Natick, MA, USA) with the DOMFluor toolbox (http://www.models.life.ku.dk). The appropriate number of components was determined primarily based on the three diagnostic tools including residual analysis, core consistency and visual inspection of spectral shapes of each component, which are widely used by other similar studies [20,21]. The components extracted by PARAFAC represent groups of the organic fractions that exhibit similar fluorescence properties. The component scores indicate the relative concentration of the groups, not the actual concentration of a particular fluorophore. However, it is typically assumed that the scores are proportional to the concentrations of the different components [20]. In this study, the final component scores were obtained after the dilution factors of the samples were fully considered.

### 2.4. Statistical Analyses

Regression and correlation analyses were performed using XLSTAT (Addinsoft, New York, NY, USA). Significances of the correlations in the statistics were evaluated using p-values. The total number of the data for the statistical analyses was 35.

## 3. Results and Discussion

### 3.1. General Water Quality Parameters for Organic Matter

The concentrations of BOD and COD ranged from 0.5 mg/L to 25.4 mg/L and from 1.6 to 20.6 mg/L, respectively (Table 1).

As expected, the highest and the lowest concentrations were observed for the WWTP effluent and for the samples collected from the uppermost sites of the watershed, respectively. The concentrations of BOD exceeded the COD concentrations for the sampling sites near the WWTP, indicating that the BOD measurements may be influenced by nitrogenous BOD in sewage due to the presence of ammonia [22]. The organic matter-related water quality was substantially recovered after joining the effluent (St. 17) from the Daecheong reservoir, presenting the average BOD and COD concentrations of 2.3 mg/L and 7.6 mg/L, respectively. Total nitrogen concentrations in the watershed exhibited similar spatial variations, ranging from 1.8 mg/L to 20.0 mg/L. The levels of the three water quality parameters tend to increase downstream at the sites located upstream from the WWTP, suggesting that, aside from the WWTP effluent, the river water quality may be deteriorated by various uncontrolled pollution loads from the residential areas [23]. Additional work including the measurement of the specific pollutant loads into the main channel is required to fully justify the reasons.

### 3.2. Fluorescence EEM Characteristics

**Figure 2.**
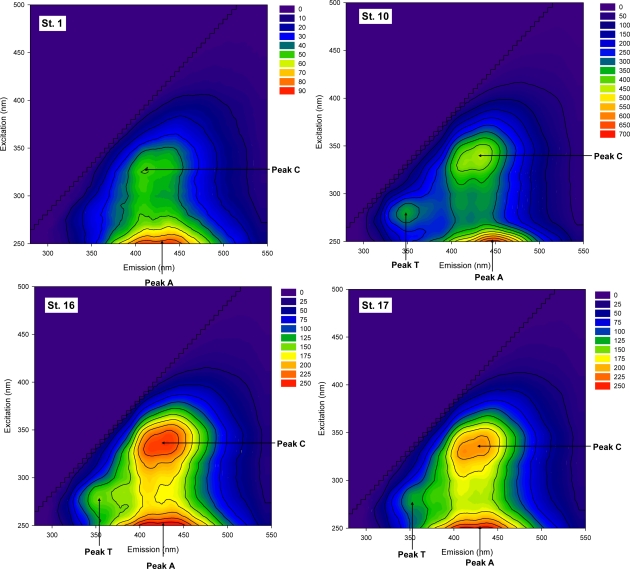
Fluorescence EEM spectra of the representative samples.

Three distinctive peaks can be identified from the fluorescence EEM of the collected samples (Figure 2). The tryptophan-like peak located at the excitation/emission wavelengths of 275 nm/340 nm (Peak T) was the most pronounced for the WWTP effluent samples (St. 10). The tryptophan-like substances may be associated with freely dissolved aromatic amino acid as well as the molecules bound with proteins and humic substances [10,24]. The peak has been used as an indicator of anthropogenic activities related to organic pollution. Many previous studies revealed a high association of the EEM peak with the presence of bioavailable and labile organic substrates and/or the product of microbial or algal activities [10,25–27]. Two types of humic-like EEM peaks were also found for all the collected samples (Figure 2). The peaks were located at the excitation/emission wavelengths of 250 nm/400–450 nm (Peak A) and 330–340 nm/350–400 nm (Peak C). The two peaks result from the presence of both carbon-carbon double bonds and aromatic carbon bonds in fulvic acid-like and/or humic-acid-like components. The relative ratio has been used as a tracer to distinguish between coastal and oceanic samples [28]. In general, higher relative ratios of the humic-like peaks to the tryptophan-like peak have been observed for river samples with low levels of organic pollution [10]. In this study, the fluorescence intensities of all the three EEM peaks exhibited an increasing trend with a higher degree of organic pollution (*i.e.*, higher BOD and COD concentrations). However, the relative peak ratios of the humic-like to the tryptophan-like fluorescence (*i.e.*, Peak A to Peak T or Peak C to Peak T) were lower for the sites near the WWTP and downstream of the WWTP compared to the upstream sites (Table 2). Our results suggest that although all the three EEM peaks tend to increase with the degree of organic pollution, Peak T may serve as a more sensitive surrogate for the point source pollution. Thus, the ratio of Peak A to Peak T or Peak C to Peak T appears to be useful as an evaluation index for the impact of treated sewage on urban rivers [8].

### 3.3. PARAFAC Components from EEM Data

**Figure 3.**
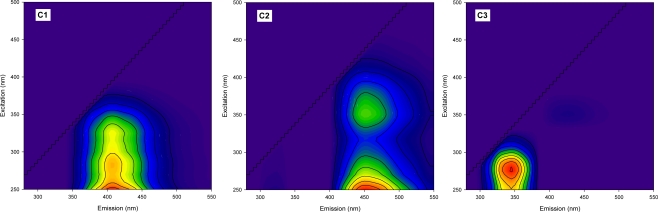
Contour plots of the three PARAFAC components decomposed from our samples.

The diagnostic tools used for this study revealed that three components are adequate for the PARAFAC model. In other words, all the fluorescence EEM data could be successfully decomposed into a three-component model by the PARAFAC analysis. Figure 3 shows each contour plots of the three PARAFAC components. A single peak, located at the excitation/emission wavelengths of 250 nm/405 nm, was observed for the contour of component 1 (C1) whereas component 2 (C2) showed two peaks at 250 nm/450 nm and at 350 nm/450 nm of the excitation/emission wavelengths. C1 and C2 may be associated with the presence of humic-like substances because the peak locations are very similar to those of Peak A and Peak C previously observed in our EEM data. However, the two fluorescent components were different from each other, not only in the number of the peaks, but also in their locations. C1 possesses a peak at a shorter emission wavelength than C2, suggesting that, despite their similar origins, the fluorophores responsible for component 1 may contain less conjugated and less condensed structures than C2 [29]. The blue-shifting of fluorescence spectra is also related to a lower degree of the humification in the DOM samples. Thus, component 1 can be interpreted as a group of less humified fluorescent substances with low molecular weights [11]. Although C1 appears to exhibit a single peak, the width of the excitation maximum (50–100 nm) indicates that it may be a mixture of fluorophores. The high ratio of Peak A to Peak C for C1 may be associated with photobleaching and/or new production of fluorophores having high excitation in Peak A region [30]. Component 3 (C3) resembles the tryptophan-like fluorescence EEM pattern, indicating the origin of the fluorophore group may be related to microbial derived amino acid and/or protein-like substances. The observed spectral characteristics of the components determined in this study generally agree with those reported in the literature based on different aquatic environments [31–33]. For particular, our PARAFAC component patterns were very consistent with a recent study by Zhang *et al.* [16], who characterized DOM in Lake Tianmuhu and its catchment basin in China using PARAFAC modeling of the fluorescence EEM data. In their study, C1 has been explained as microbial humic-like components as a result of the microbial transformation from terrestrially-derived organic matter, and C2, as a mixture of the traditional humic-like fluorescence peaks [16].

The score values of all the components exhibited a similar trend in the variation with the concentrations of BOD and COD. The most dramatic change was observed for C3 (Table 2), suggesting that the component can be used as a good tracer for the degree of organic pollution. For the sites of the tributaries (*i.e.*, Daejeon River and Yudeung River) located upstream of the WWTP, the ratio of C1 to C2 showed an increasing trend downstream, indicating the influent rivers may continuously receive the input of less humified organic matters (*i.e.*, microbial humic-like) before they join the main channel.

**Figure 4.**
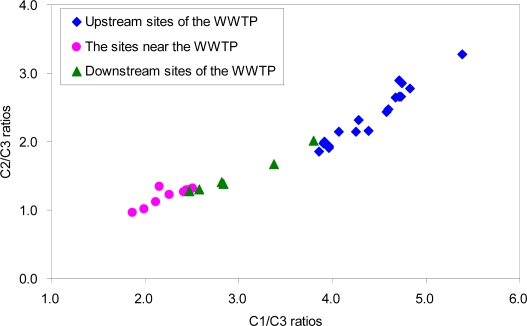
A plot of the C2/C3 ratios against the C1/C3 ratios for the discrimination of the sampling sites (Site 17 is excluded).

A plot of the ratios of C1/C3 and C2/C3 showed spatial differences depending on the location of the sampling sites (Figure 4). For example, the sites near the WWTP exhibited relatively low ranges for the two ratios while the highest ranges were observed for the upstream sites of the WWTP. The downstream sites of the WWTP corresponded to the intermediate range. Our results suggest that C3 is highly associated with the source of the WWTP effluent. In contrast, C1 and C2 appear to reflect the characteristics of DOM in the upper sites, in which terrestrial humic-like and microbially transformed organic materials may be relatively more enriched.

The three components were all highly correlated each other (r > 0.970; p < 0.001), suggesting that the original sources of the fluorescence components are not much different from each other. As expected, the highest correlation was observed between C1 and C2 (r = 0.992).

### 3.4. Correlations between General Organic Matter Parameters and PARAFAC Results

Correlation coefficients between some selected spectroscopic indices and water quality parameters including BOD, COD, and TN were calculated and are compared in Table 3. UV absorption values at the wavelengths of 220 nm and 254 nm, which are designated as UV_220_ and UV_254_, respectively, were chosen as non-fluorescence indices because nitrate ions are known to strongly absorb UV light at a wavelength of 220 nm [15], and UV absorbance at the wavelengths between 250 nm and 280 nm has been widely used to monitor BOD and COD concentrations in river and wastewater samples [34,35]. The estimation capability of each index was evaluated using Spearman’s rho as well as Pearson’s r values because most of our water quality data is highly skewed and distributed in low concentration ranges.

In this study, all the selected spectroscopic indices showed significant correlations with BOD, COD and TN concentrations (p < 0.001). As expected, TN concentrations exhibited the highest correlation with UV_220_ based on Spearman’s Rank correlation coefficient. However, such a good correlation was not very pronounced considering Pearson r values. For example, the r value based on UV_220_ was even lower than the value calculated between UV_254_ and TN concentrations, which did not agree with the common observation of the little UV absorption of nitrate at a wavelength of 254 nm. Our result suggests that Spearman’s Rank correlation coefficients should be included in evaluating the estimation indices for a particular water quality parameter.

**Figure 5.**
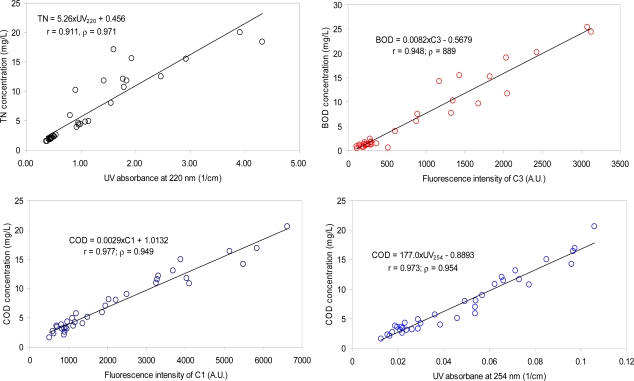
Correlations between selected spectroscopic indices (UV absorption indices or PARAFAC components) and water quality parameters (TN, BOD, and COD).

In this study, the difference of UV absorbance at the wavelength between 220 nm and 254 nm, UV_220–254_, did not enhance the estimation capability for TN concentrations (Table 3; Figure 5). The two types of the correlation coefficients (both Pearson correlation and Spearman’s Rank correlation) between TN and UV_220–254_ were both lower than those obtained based on UV_220_, indicating that the removal of the interference from the presence of UV-absorbing organic components did not result in the improvement of UV_220_ estimation capability for TN. The failure of the effort may be attributed to similar trends of the variations in the organic and the nitrogen pollution, and/or similar sources of the two types of the pollution in the watershed. For example, the typical sources of nitrogen pollution in urban areas may include fertilizer use on lawns, WWTP discharge, unattended sewage disposal, and leaks from sewer lines [36], which can also be considered as organic pollution sources. The result also suggests that nitrogen species other than nitrate ions (e.g., ammonia and organic nitrogen) may be present in an appreciable amount in the river samples.

For BOD estimation, all the PARAFAC components presented higher correlation coefficients than the absorbance-based indices, suggesting that fluorescence measurements are a superior monitoring tool for biodegradable organic matters in urban rivers. C3 exhibited the highest correlation coefficient with BOD among the PARAFAC components (Table 3; Figure 5), suggesting that microbial derived amino acid and/or protein-like substances may be good indicators for labile and biodegradable organic matters in typical urban rivers. Our results are consistent with many prior reports, in which amino acid-like or protein-like fluorescence characteristics were highly correlated with BOD concentrations in rivers [1,25,37].

UV_254_ showed the highest estimation capability for COD among the selected indices based on the Spearman’s Rank correlation coefficients [38] while the Pearson’s correlation coefficient was the highest with C1 (Table 3). C3 also presented good correlation coefficients with COD (r = 0.977), suggesting that the protein-like component and microbial humic-like (or less humified) organic matter may constitute a dominant fraction of the total organic matter in the watershed. Our results are in contrast with other river samples not much affected polluted by sewage wastewater [16], in which COD concentrations are highly correlated with humic-like components but not with the protein-like component. Based on our individual estimation indices showing the highest correlation coefficients with TN, BOD, and COD, the regression equations are suggested as TN = 5.26 × UV_220_ + 0.456, BOD = 0.0082 × C3 + 0.5679, COD = 0.0029 × C1 + 1.0132, and COD = 177.0 × UV_254_ – 0.8893, respectively.

### 3.5. Improvement of the Estimation of TN Using Multiple Regression Analysis

**Figure 6.**
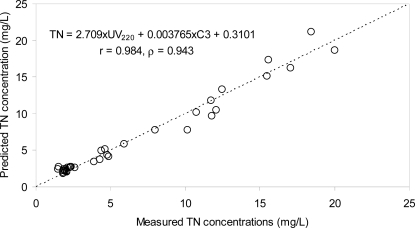
Correlations between the measured TN concentrations and the predicted values by multiple regression method.

Our previous observation of the close association between TN concentrations and the PARAFAC components (particularly C1) implies that the UV_220_ estimation capability for TN concentrations may be improved by using a multiple regression analysis based on the fluorescence indices. In this respect, a multiple regression equations based on a PARAFAC component and UV_220_ was established, and the associated correlation coefficients were calculated. Compared to the previous regression method based on a single index, the multiple regression analysis showed the enhancement of the estimation (*i.e.*, the increase in the correlation coefficients) (Figure 6).

For example, the deviation of the data points from the regression line became much less pronounced after the multiple regression analysis was applied (Figure 6). The final equation was TN = 2.709 × UV_220_ + 0.003765 × C3 + 0.3101, and the correlation coefficients were 0.984 and 0.943 for Pearson’s r value and Spearman’s rho value, respectively, exhibiting the enhancement of the estimation precision compared to the single linear correlations.

Although the fluorescence data obtained here are based on data from a standard laboratory instrument, we expect that our proposed procedure and the methodology will be useful for developing *in situ* real-time monitoring of TN, BOD, and COD concentrations in typical urban rivers. Many handheld fluorometers with various scanning function are already available, and fluorescence sensing devices are easy to make in different sizes and at a desirable level of the signal-to-noise ratio according to the purpose of the operator. More future work should be undertaken to prove successful applications of our results to *in situ* real-time monitoring techniques. For example, *in-situ* software programs to directly link the measurement data with PARAFAC modeling need to be developed. It should be noted that the regressions developed here are based on only a limited number of the samples collected during the dry season. For more successful applications, rain sampling events should be considered so that water quality and organic matter characteristics examined can be fully representative of a typical urban river.

## 4. Conclusions

Three components were successfully identified by PARAFAC modeling from the fluorescence EEM data of the samples collected from a typical urban river, which is influenced by the effluent of a WWTP and various urban anthropogenic activities. The components represented the groups of the fluorophores representing protein-like (C3) and two humic-like organic substances (C1 and C2). UV_220_, UV_254_, and the score values of the three identified PARAFAC components were chosen as the estimation indices for TN, BOD, and COD concentrations of the river samples. Among the selected indices, UV_220_, C3 and C1 exhibited the highest correlation coefficients with TN, BOD, and COD concentrations, respectively. For TN, multiple regression analysis using the equation, 2.709 × UV_220_ + 0.0038 × C3 + 0.3101, demonstrated the enhancement of the estimation capability. The corresponding Pearson’s r values and Spearman’s rho values were 0.98 and 0.94, respectively. The selected spectroscopic indices and the associated methodology proposed here are expected to be usefully employed for the development of real-time monitoring techniques.

## Figures and Tables

**Table 1.** Monitoring data of turbidity, SS, BOD, COD, and TN concentrations for the Gap River watershed (September/October) ^a^.

**Sites**	**Location**	**Turbidity (NTU)**	**SS (mg/L)**	**BOD (mg/L)**	**COD (mg/L)**	**TN (mg/L)**
St. 1		2.5/1.4	2.3/1.6	0.7/1.1	3.2/2.6	1.9/1.9
St. 2		1.7/1.4	1.0/1.4	0.8/1.4	3.3/3.1	1.8/2.0
St. 3		5.6/3.4	6.0/3.4	1.3/1.8	4.0/4.2	2.3/2.4
St. 4		1.6/1.2	1.0/1.6	0.5/0.9	2.7/1.6	2.0/1.8
St. 5	Upstream sites of the WWTP	1.2/1.5	0.5/1.6	1.0/1.2	3.6/2.3	2.0/1.9
St. 6		0.8/0.7	0.7/1.0	0.8/1.3	3.8/2.1	3.9/4.9
St. 7		1.5/1.0	1.8/1.2	1.2/1.6	4.3/3.6	4.3/4.8
St. 8		1.7/1.5	1.7/2.8	1.3/1.7	3.4/3.1	2.1/2.6
St. 9		4.3/4.1	4.8/5.4	1.4/2.4	5.7/4.9	2.3/2.1

St. 10		2.1/2.1	3.8/4.8	24.4/25.4	16.9/20.6	15.6/17.1
St. 11		3.0/ND ^b^	4.5/ND	19.1/ND	11.7/ND	11.8/ND
St. 12	Near the WWTP	2.8/2.7	3.5/4.6	7.5/10.3	8.0/10.9	5.9/10.2
St. 13		1.6/1.7	2.5/4.2	20.2/11.7	14.2/16.4	18.4/20.0
St. 14		2.6/2.0	5.5/4.9	9.7/15.2	10.8/15.0	12.5/15.5

St. 15	Downstream sites of the WWTP	4.2/2.7	8.8/5.8	7.8/15.5	11.5/13.1	10.7/12.1
St. 16		4.1/3.7	7.0/6.4	6.1/14.2	9.0/12.1	8.0/11.8

St. 17	Discharge of a dam reservoir	2.2/2.4	2.5/3.4	1.5/1.3	5.9/5.1	1.5/1.5

St. 18	Downstream sites of the WWTP	3.3/2.8	5.3/5.6	0.6/4.0	7.0/8.1	4.4/4.6

a One sample was taken per site for each sampling event (09/25/2005, 10/18/2005);

b Not determined due to the failure of the sampling.

**Table 2.** Selected spectroscopic characteristics of the river water for this study (September/October) ^a^.

**Sites**	**Ratio of Peak A to Peak T**	**Ratio of Peak C to Peak T**	**A_220_ (l/cm)**	**A_220_–A_254_ (l/cm)**	**C1 (A.U.)**	**C2 (A.U.)**	**C3 (A.U.)**
St. 1	4.39/3.30	2.41/1.88	0.44/0.45	0.41/0.43	888/899	546/475	188/221
St. 2	4.26/3.17	2.37/1.76	0.42/0.44	0.40/0.41	925/950	557/487	195/243
St. 3	4.17/3.26	2.27/1.76	0.51/0.47	0.47/0.44	1,355/1,164	760/567	286/293
St. 4	5.18/4.03	2.88/2.27	0.46/0.41	0.44/0.39	589/503	358/284	109/107
St. 5	4.31/3.39	2.30/1.95	0.46/0.41	0.44/0.39	689/623	396/337	142/145
St. 6	4.02/3.77	2.27/2.00	0.92/1.14	0.90/0.39	805/888	428/436	176/202
St. 7	3.79/3.35	2.02/1.75	0.96/1.08	0.93/1.12	964/1125	486/540	227/284
St. 8	4.04/3.08	2.22/1.63	0.46/0.54	0.44/1.06	700/821	397/414	150/210
St. 9	4.08/3.01	2.28/1.71	0.50/0.47	0.46/0.52	1,184/1,091	636/525	257/282
St. 10	1.35/2.07	1.06/1.32	1.93/1.59	1.83/2.1	5,853/6,622	3,006/4,151	3,126/3,080
St. 11	1.40/ND ^a^	1.19/ND	1.41/ND	1.34/ND	4,036/ND	2067/ND	2,031/ND
St. 12	2.03/1.91	1.28/1.32	0.80/0.90	0.75/0.90	2,210/3,247	1,152/1,702	888/1343
St. 13	1.80/1.85	1.33/1.74	4.32/3.92	4.23/3.92	5,485/5,133	2,960/2,691	2,425/2,046
St. 14	2.00/1.95	1.37/1.45	2.46/2.92	2.39/2.92	4,096/3,872	2,168/2,041	1,677/1,827
St. 15	1.87/1.93	1.51/1.67	1.79/1.77	1.72/1.77	3,279/3,680	1,685/1,863	1,325/1,428
St. 16	2.26/2.18	1.55/1.78	1.54/1.84	1.49/1.84	2,486/3,301	1,212/1,644	877/1,170
St. 17	4.63/4.33	3.93/2.93	0.38/0.36	0.32/0.36	1,860/1,480	1,079/822	361/303
St. 18	3.22/2.65	2.19/1.79	0.99/0.95	0.94/0.95	1,939/2,027	1,031/1,001	511/600

a One sample was taken per site for each sampling event (09/25/2005, 10/18/2005);

b Not determined due to the failure of the sampling.

**Table 3.** Correlation coefficients ^a^ (Pearson r values and spearman rho values) between selected spectroscopic indices and some water quality parameters (n = 35).

	**TN**	**BOD**	**COD**
UV_220_	0.911 ^b^	0.706	0.770
	0.971 ^c^	0.720	0.750
UV_254_	0.914	0.892	0.973
	0.747	0.813	0.954
UV_220–254_	0.905	0.696	0.759
	0.968	0.704	0.741
C1	0.951	0.948	0.977
	0.806	0.861	0.949
C2	0.927	0.938	0.967
	0.772	0.823	0.937
C3	0.950	0.948	0.977
	0.806	0.889	0.936

a Significant levels p values are all lower than 0.001 (p < 0.001);

b Pearson r values;

c Spearman rho values.
